# Effects of intermittent hypoxia and whole-body vibration training on health-related outcomes in older adults

**DOI:** 10.1007/s40520-023-02655-w

**Published:** 2024-01-27

**Authors:** Rafael Timón, Adrián González-Custodio, Narcis Gusi, Guillermo Olcina

**Affiliations:** Facultad de ciencias del deporte, Universidad de Extremadura, Av/Universidad s/n, 10003 Cáceres, Spain

**Keywords:** Hypoxia, Whole-body vibration, Inflammatory biomarkers, Bone, Muscle, Fitness

## Abstract

**Background:**

Aging is associated with a health impairment and an increase of the vulnerability of the older people. Strength training under intermittent hypoxic conditions has been shown to have therapeutic effects on individual’s health.

**Aims:**

The aim of this study was to investigate the effects of a combined intermittent hypoxia (IH) and whole-body vibration (WBV) training program on health-related outcomes in older people.

**Methods:**

A total of 60 adults (over the age of 65) voluntarily participated in an intervention that lasted 20 weeks (three 30-min sessions per week). The participants were divided into four experimental groups subjected to different environmental conditions (IH vs normoxia) and exercise (non-exercise vs WBV). Functional fitness, body composition, metabolic parameters, inflammatory biomarkers, and bone turnover were evaluated before and after the intervention. A multifactorial ANOVA with repeated measures was performed to explore differences within and between groups.

**Results:**

The results showed that IH and WBV had a positive synergistic effect on inflammatory parameters (CRP and IL-10), bone formation biomarker (PINP), and body composition (muscle and bone mass).

**Conclusion:**

In conclusion, a combined IH and WVB training could be a useful tool to prevent the deterioration of health-related outcomes associated with aging.

*Clinical trial registration* NCT04281264. https://clinicaltrials.gov/.

## Introduction

Life expectancy in developed countries has increased steadily in recent years, leading to aging of the world’s population. Aging is a degenerative process that causes a progressive decrease in bone mass, muscle mass and a loss of functionality, decreasing physiologic resilience, and increasing vulnerability [1].

Physical exercise has been proposed as an effective tool to protect health and cope with the impairment of physical and cognitive abilities of older people [2–4]. Specifically, it has been concluded that strength training plays an important role for older people because it decreases chronic inflammation and attenuates intramuscular adipose infiltration, and increases muscle cross-sectional area, improves bone density, cardiovascular health, metabolic health and insulin sensitivity, as well as amino acid absorption and protein synthesis [5, 6]. Anyway, it has been suggested that resistance training programs must be tailored to each older adult according to age, sex, and other individual aspects [7].

Traditional methods of strength training (free weights and machines) are not very viable options for most seniors due to time constraints, a high perceived difficulty and complexity of the technique [8], so it is necessary to research on time-efficient resistance training strategies that are practical and effective. The whole-body vibration (WBV) has proven to be a safe, accessible, and effective tool to counteract age-related declines and improve health-related outcomes (body composition, functional fitness, bone and cardiovascular health) in weak individuals and older adults [9, 10]. Different mechanisms have been proposed to explain the improvements achieved with the WBV training. WBV induces predominance of anabolic hormones and increased energy expenditure that may lead to an increase in lean mass and reduction in fat mass [11]. Likewise, WBV requires an important response from the muscle and bone tissues to dampen the energy caused by oscillatory actions, stimulating the osteogenic response, and increasing bone mass [12]. The reflex muscle contraction induced by WBV causes a tonic vibration reflex and a more efficient proprioceptive feedback loop that increases force production and functional capacity [11]. In addition to this, WBV could act on inflammatory status due to its capacity for stimulating nerve sensors that promote systemic responses, regulate the immune system, and reduce the circulatory levels of inflammatory cytokines [13, 14]. However, current reviews have shown conflicting results concluding that WBV does not seem to simultaneously influence bone and muscle health in older people [15]. The type of intervention (i.e., frequency, amplitude) as well as the duration of training (i.e., total volume, number of sessions) could explain this discrepancy in the results [12, 16]. In addition to this, it has been stated that mechanotransduction could fluctuate at different regions of the body due to the nonlinear musculoskeletal system and the different body position used [17], so the conclusions reached could be different depending on the training routines, or even the type of assessments carried out.

Intermittent hypoxia (IH) has also been proposed as a new therapeutic tool to mitigate age-related impairments [18, 19]. IH allows modulating and stabilizing the hypoxia-inducible factor-1 alpha (HIF-1α), which is involved in the expression of factors related to angiogenesis, osteogenesis, lipolysis, and regulation of the inflammatory response [20, 21]. Senior adults have elevated circulating levels of pro-inflammatory cytokines and C-reactive protein (CRP) that could lead to cardiovascular and metabolic diseases [22], as well as loss of muscle and bone mass [23, 24], causing a drop in functional fitness and personal autonomy. However, resistance training performed under hypoxic environment in older adults enhanced body composition and physical fitness [25], decreased the low-grade chronic inflammation [26], and led to beneficial effects on bone turnover biomarkers [27]. Park et al. [25] concluded that hypoxic training during 12 weeks (3 days/week) caused a significant loss of body weight and % body fat, and an increase in fat-free mass and physical fitness (chair stand, pegboard, one leg standing and tandem test). Timon et al. [26] showed that 24 weeks of resistance training under IH produced a significant decrease of CRP and IL-8, and an increase of IL-10 concentrations. Likewise, 24-weeks of normobaric hypoxic conditioning caused a 59% drop in levels of C-terminal telopeptide of collagen (b-CTX), which is a bone resorption biomarker that would indicate an improvement in bone turnover and bone health [27].

Based on this background, the combination of IH and WBV could have a beneficial synergistic effect on health-related outcomes of older people. In this vein, our research group has previously shown that 18 weeks of WBV training (12.6 Hz in frequency and 4 mm in amplitude) combined with hypoxic stimuli (16.1% of fraction of inspired oxygen, FiO_2_) in older adults over 65 years of age caused a slight but significant increase in bone mineral density after 36 intervention sessions [28], although without changes in both leg muscle mass and functional mobility [29].

Therefore, the aim of this study was to investigate the effects of a combined training program of IH and WBV on health-related outcomes (body composition, functional fitness, metabolic parameters, inflammatory and bone turnover biomarkers) in older adults. We hypothesized that IH and WBV training would have positive synergistic effects on the health of older adults, leading to a decrease of CRP, metabolic parameters and fat mass, and an improvement of anti-inflammatory IL-10, bone turnover biomarkers, muscle mass, muscle strength, and functional capacity.

## Materials and methods

The research design and the protocol used in the research were registered in the international registry of clinical trials of the U.S. National Institutes of Health (https://clinicaltrials.gov/), with the identifier NCT04281264.

### Participants

A total of 60 older people, over 65 years old, were recruited in the study. The personal data of participants who completed the study are shown in Table 1 (age, BMI, score in PAR questionnaire, and daily intake of kilocalories, vitamin D and calcium). Organizations of retired people, nursing homes, and senior universities were contacted to inform them about the research study and recruit volunteers. Participants were selected after a screening visit, in which the following inclusion criteria had to be met: (1) women and men over 65 years old, (2) absence of participation in any other type of intervention based on physical exercise in the past 3 months, (3) not having been 1500 m above sea level during the past 3 months, (4) no current medical condition that was not compatible with WBV training, (5) no disorders of the musculoskeletal system or cardiometabolic disease (except treated hypertension), (6) free of anti-inflammatory drugs or medication that affects the bone system, (7) consumption of no more than two alcoholic beverages per day, and (8) non-smoker.

**Table 1:** Characteristics of the participants at baseline

	Age	BMI	PAR-Q	Kcal/ day	Vit D (IU/day)	Calcium (mg/day)
Normoxia (*n* = 14)	70.8 ± 4.3	27.0 ± 2.1	2.7 ± 0.9	2158.3 ± 206.1	368.3 ± 20.5	918.5 ± 63.4
Hypoxia (*n* = 13)	69.7 ± 3.1	27.7 ± 3.6	2.7 ± 0.6	2087.4 ± 119.3	354.2 ± 22.3	909.3 ± 53.2
Normoxia + WBV (*n* = 13)	69.6 ± 4.3	25.8 ± 2.9	2.9 ± 0.8	2186.0 ± 128.6	340.5 ± 25.9	911.4 ± 54.4
Hypoxia + WBV (*n* = 12)	69.9 ± 4.7	26.2 ± 1.3	2.8 ± 0.4	2024.5 ± 150.6	332.1 ± 39.5	920.5 ± 47.3

*BMI* body mass index; *PAR-Q* Physical Activity Rating Questionnaire; *WBV* whole-body vibration

The sample size was calculated using G* Power software (version 3.1) to achieve a statistical power of 0.80, with a significance level of 0.05 and a medium effect size (*f* = 0.25). The calculation resulted in a total sample size of 48 individuals.

### Experimental design

This was a pre–post single-blind randomized controlled study, although balancing the experimental groups by sex. The eligible participants were divided into four experimental groups: normoxia exposure (NOR; *n* = 14, 6 males and 8 females), hypoxia exposure (HYP; *n* = 13, 7 males and 6 females), normoxia + WBV (NWVB; *n* = 13; 6 males and 7 females), and hypoxia + WBV (HWBV; *n* = 12, 5 males and 7 females). The participants were blinded to the hypoxic or normoxic condition. The intervention lasted 20 weeks, three 30-min sessions per week with 1 rest day between sessions. Group adherence to training was set at 75% attendance. Participants were asked to continue with their usual lifestyle and diet throughout the intervention, and they were allowed to continue using their usual medication. However, individuals who had unstable medical conditions or new medications within the data collection period were excluded. Outcomes were measured at baseline before the intervention (Pre) and after the intervention (Post). The research was approved by the Bioethics Committee of the university (Ref: 651/2018) and was carried out respecting the ethical principles established in the Declaration of Helsinki. The participants signed an informed consent and could leave the research at any time.

### Training protocol

The intervention was performed by certified sports scientists. The training program lasted 20 weeks, three 30-min sessions per week, with 1 rest day between sessions. The duration of the intervention session was the same for all groups. Before starting the training programs, two familiarization sessions were held with the participants to learn the technique of the exercises on the vibration platform and to experience the hypoxic environments.

Depending on the experimental group, training was performed under conditions of normoxia or normobaric hypoxia. The hypoxic intervention took place inside a hypoxia chamber (CAT 310, Louisville, USA) installed in the laboratory (located at 459 m above sea level). The hypoxic environment was produced by a hypoxic generator (CAT 12, Colorado Altitude Training, USA), driving an air stream of up to 100 L/min. The hypoxic groups were at a simulated altitude of 2500 m above sea level (FiO_2_ of 16.1%), regularly monitoring oxygenation with an electronic device (HANDI + , Maxtec, Salt Lake City, USA). The simulated altitude was calculated according to the chart and guidelines provided by the hypoxic generator manufacturer. For safety reasons, a finger pulse oximeter was used to ensure that blood oxygen saturation (SpO_2_%) did not fall below 85%. The normoxic groups were also inside the hypoxia chamber, but with the generator driving air with a FiO_2_ of 21% (ambient air concentration). Furthermore, to blind participants, hypoxia generator was covered to prevent participants from visually identifying the normoxic or hypoxic conditions.

WBV training, whether in conditions of hypoxia or normoxia, was performed on a vibrating platform oscillating (Galileo Fitness, Novotec Medical, Germany). No information about the potential effects of the vibration treatment was given to the participants to minimize psychological effects. The duration of the sessions was 30 min, which included a 5-min warm-up with body mobilization and dynamic stretching, a 20-min main part of WVB and a 5-min cool down with stretching exercises and relaxation. The vibratory stimulus used during the first 10 weeks was at the frequency of 18 Hz and a peak-to-peak displacement of 4 mm, with an acceleration of 2.6 g; whereas for the second 10 weeks, the frequency increased to 22 Hz, the peak-to-peak displacement remained constant at 4 mm, and the acceleration increased to 3.8 g. Participants performed the protocol without shoes to avoid any vibration attenuation. Four different exercises were performed with four sets of 30 s of WBV for each, with a break of 60 s between sets. The exercises carried out were as follows: (1) static squatting position at 120° (measured by goniometer), upright trunk, and simultaneously performing biceps curl (12–15 reps.) with elastic band attached to the feet; (2) lunge with one leg positioned forward with knee bent (90°) and foot flat on the vibration platform while the other leg is positioned behind on the floor, simultaneously performing standing row with elastic band (12–15 reps). Legs were alternated in each series; (3) glute bridge exercise with feet on the vibration platform and the upper back resting on the floor, creating a straight line from knees to shoulders; (4) front plank, with feet on the vibration platform and forearms resting on the floor.

Unlike the NWBV and HWVB groups, the NOR and HYP groups did not perform any exercise and were seated (reading, watching the mobile phone or tablet) for 30 min.

### Measurements

Measurements were made 3 days before starting the intervention (pre-values) and 3 days after finishing the last session of the intervention (post-values). The assessment tests were performed in the following order: Physical Activity And Dietary Questionnaire, fasting blood collection, anthropometry, body composition, and functional fitness test.

On their first visit to the laboratory, participants answered an adapted version of the Physical Activity Rating Questionnaire (PAR-Q) to evaluate their level of physical activity [30], with scores between 0 (lowest level) and 7 (highest level). Likewise, the kilocalories, vitamin D, and calcium intake of the participants were estimated using a 7-day diet inventory, which was analyzed using the diet software Nutriber (Nutriber v1.1.1, Funiber, Barcelona, Spain).

Then a blood extraction was performed after a minimum of 10 h of overnight fasting. Blood samples were taken from the antecubital vein by one experienced nurse using vacutainer tubes containing gel separators for serum analytics. After 10 min of centrifugation at 1790 g (relative centrifugal force), serum was extracted and injected into micro-centrifuge tubes. The determination of glucose, triglycerides, and total cholesterol (CHO) was carried out within a maximum period of 1 h after extraction. The determination of these metabolic parameters was performed with an automatic dry-chemistry analyzer system (Spotchem EZ SP-4430; Arkray, Inc., Kyoto, Japan). The calibration of the device was checked daily through indicated reagent cards, according to the manufacturer’s recommendation. The rest of serum micro-centrifuge tubes were stored at − 80 °C until the determination of CRP, interleukin 10 (IL-10), interleukin 8 (IL-8), N-terminal propeptide of type I procollagen (PINP), and C-terminal telopeptide of collagen (b-CTX). Interleukins were analyzed using validated ProcartaPlex Multiplex immunoassay kits (Invitrogen, Bender Med Systems GmbH, Wien, Austria) following its standard operating procedure, which is based on Luminex technology (Bioplex 200, Bio-Rad, Hercules, USA). The intra-assay coefficient of variability (CV) was 8.5% for IL-8 and 3.1% for IL-10. Serum CRP, PINP, and b-CTX concentrations were determined by colorimetric sandwich of enzyme-linked immunosorbent assay kits with an ELISA microplate reader (SpectraMax PLUS 384, Molecular Devices, San Jose, USA), following the manufacturer’s instructions. The intra-assay coefficient of variability was 8.8% for CRP and < 8% for both PINP and b-CTX.

Functional fitness was evaluated by the same technician in the morning (10:00–12:00 a.m). Participants had to perform five tests included in the Senior Fitness Test (SFT) [31]: chair stand test (to assess lower body strength); arm curl test (to measure upper body strength); 6-min walking test (to assess aerobic endurance); chair sit and reach test (to assess lower body flexibility); and 8-foot up and go test (to assess agility and dynamic balance). SFT is considered a reliable instrument to assess functional capability in older adults (≥ 60 years of age) [32]. The strength of the hand and forearm muscles was measured using a handgrip dynamometer (Baseline Smedley, Model 12-0286, White Plains, USA). Participants were instructed to grip the device with maximum strength for 3 s. They performed two test trials with the dominant hand and the mean was used for data analysis. In addition, the front plank test was performed to evaluate the endurance of the core stabilizing muscles. Participants started with the upper body supported off the ground by the elbows and forearms, and the legs straight with the weight taken by the toes. The hip was lifted off the floor creating a straight line from head to toe. The test was over when the individual was not able to hold the back straight and the hip was lowered. The score was the total time completed.

Body mass and height were measured using a portable stadiometer (Seca 213, Hamburg, Germany), and body mass index (BMI) was calculated from the ratio of mass/height^2^ (kg/m^2^). Muscle mass, fat mass, and bone mineral content (BMC) were calculated using dual-energy X-ray absorptiometry (DXA, Norland Excell Plus, Norland Inc., Fort Atkinson, USA). The standard CVs was 0.9% for muscle mass, 1.4% for fat mass, and 1.3% for BMC. The same technician performed all the scans, which were analyzed by a graphical user interface to Windows operating system.

During training sessions, SpO_2_% and rate of perceived effort (RPE) were monitored once a week randomly between participants measured. Measurements were taken 2 min after the last exercise of the session. The mean values obtained from the 20 weeks that the intervention lasted were calculated. SpO2% was measured in duplicate using a pulse oximeter (WristOx 3100; Nonin, USA). RPE was obtained by showing a graphical scale to participants with a category-ratio 10 scale, ranging between 1 (extremely easy) and 10 (extremely hard).

### Statistical analysis

Statistical analyses were performed using the Statistical Package for Social Sciences (SPSS, version 26.0, Chicago, IL, USA). The Shapiro–Wilk test was applied to verify normal distribution of data, and Levene’s test was used to assess the homogeneity of variance. A multifactorial ANOVA with repeated measures was performed to explore within-group (pre–post values) and between-group (Environment factor and WBV factor) differences. Interaction effects were tested using the multivariate criterion of Pillai’s trace. The exploration of the within-group differences was made using Bonferroni post hoc when required, using the SPSS syntax commands. The confidence interval (CI) for the difference has been shown to provide an estimate of the absolute difference in means of variables of interest. The effect size (ES) was also calculated. The magnitude of effect was interpreted using partial eta squared (*η*_*p*_^2^) as follows: > 0.01 small; > 0.06 moderate and > 0.14 large [33]. The significance level was set at *p* ≤ 0.05, with a confidence level of 95%.

## Results

A total of 52 out of 60 participants completed the whole study and their results were included in the analysis. These participants had good attendance to the sessions (over 83%). Blinding was successful as more than 60% of subjects did not guess whether they were under hypoxic or normoxic conditions.

There were eight participants who dropped out: NOR (1 × personal reasons), HYP (1 × new medication and 1 × low adherence), NWBV (1 × sickness and 1 × low adherence), and HWBV (1 × discomfort and 2 × low adherence). There were no research-related adverse effects or injuries.

No between-group significant differences were observed for control variables at baseline (see Table 1). According to reference daily intake (RDI), an insufficient vitamin D and calcium intake was observed in all groups.

The SpO_2_% values indicated that the environment during training sessions were significantly different between normoxic and hypoxic conditions (NOR: 97.3 ± 1.2; HYP: 91.1 ± 1.3; NWBV: 96.4 ± 1.4; HWBV: 91.2 ± 1.1). The RPE values were significantly higher during the WBV interventions compared with the non-exercise interventions, although without observing significant differences related to environmental conditions (NOR: 0.0 ± 0.0; HYP: 0.2 ± 0.4; NWBV: 4.2 ± 0.8; HWBV: 4.7 ± 1.6).

Table 2 shows body composition variables at baseline and after 20 weeks of intervention. Muscle mass and BMC increased significantly in HWBV, observing between-group differences in muscle mass with a moderate interaction effect of the environment factor (*F* = 4.644; *p* = 0.037; ES: 0.097), and in BMC with a large interaction effect of the WBV factor (*F* = 9.066; *p* = 0.004; ES: 0.174) and environment*WBV (*F* = 8.983; *p* = 0.005; ES: 0.173).

**Table 2:** Body composition variables before and after the intervention (mean ± SD)

	Normoxia			Hypoxia			Normoxia + WBV			Hypoxia + WBV			PrePost*Env	PrePost*WBV	PrePost*Env*WBV
	Pre	Post	95%CI	Pre	Post	95%CI	Pre	Post	95%CI	Pre	Post	95%CI	F (effect size)	F (effect size)	F (effect size)
Weight (kg)	70.2 ± 9.6	69.9 ± 9.8	− 0.76–0.65	72.8 ± 5.7	72.6 ± 4.7	− 1.14–2.04	71.3 ± 11.6	71.2 ± 11.8	− 0.03–2.22	69.4 ± 9.8	70.4 ± 9.3	− 1.03–0.34	0.820 (0.019)	0.117 (0.003)	3.546 (0.076)
BMI	27.0 ± 2.1	26.9 ± 2.4	− 0.31–0.25	27.7 ± 3.6	27.9 3.2	− 0.39–0.75	25.8 ± 2.9	25.7 ± 3.1	− 0.01–0.81	26.2 ± 1.3	26.7 ± 1.1	− 0.43–0.11	0.834 (0.019)	0.057 (0.001)	3.978 (0.085)
Muscle mass (Kg)	44.6 ± 10.2	44.1 ± 10.3	− 0.18–1.39	44.0 ± 9.5	44.4 ± 9.5	− 0.65–0.10	45.5 ± 10.4	45.6 ± 10.2	− 1.25–1.54	42.6 ± 9.9	43.6 ± 10.1*	− 2.38–1.16	**4.644 (0.097)**^**+**^	2.780 (0.061)	0.017 (0.001)
Fat mass (Kg)	23.6 ± 4.9	23.8 ± 7.7	− 2.93–2.41	26.3 ± 4.4	25.7 ± 4.1	0.07–1.42	23.2 ± 5.7	22.9 ± 5.8	0.34–0.80	24.7 ± 5.2	24.6 ± 4.8	− 1.75–1.88	0.251 (0.006)	0.103 (0.002)	0.100 (0.002)
BMC (Kg)	2.3 ± 0.4	2.3 ± 0.4	− 0.06–0.01	2.6 ± 0.4	2.6 ± 0.4	− 0.02–0.10	2.5 ± 0.4	2.6 ± 0.4	− 0.07–0.01	2.2 ± 0.7	2.4 ± 0.6**	− 0.51–0.08	1.920 (0.043)	**9.066 (0.174)**^**++**^	**8.983; (0.173)**^**++**^

Values that reach statistical significance are in bold

*Env* environment factor; *WBV* whole-body vibration factor; *BMI* body mass index; *BMC* Bone mineral content

*Within-group significant differences (pre–post). *p ≤ 0.05; **p ≤ 0.01

^+^Between-group significant differences. ^+^*p* ≤ 0.05; ^++^*p* ≤ 0.01

Blood biomarkers and metabolic parameters are presented in Table 3. At the within-group level, a significant decrease in CRP was observed in the groups that trained under hypoxic conditions (HYP and HWBV). Likewise, there was a significant increase in IL-10 in HWBV, as well as a significant increase in PINP in all groups (HYP, NWBV, and HWBV) except for the NOR group. Between-group differences showed an interaction effect of the environment factor on the variables CRP (*F* = 12.267; *p* = 0.001; ES: 0.222), IL-10 (*F* = 6.796; *p* = 0.013; ES: 0.136) and PINP (*F* = 4.071; *p* = 0.050; ES: 0.086) after intervention. A moderate interaction effect of the WBV factor was also observed in CRP (*F* = 4.137; *p* = 0.048; ES: 0.088) and PINP (*F* = 4.548; *p* = 0.039; ES: 0.096) values. Moreover, CRP (*F* = 7.789; *p* = 0.008; ES: 0.153) and IL-10 (*F* = 6.822; *p* = 0.012; ES: 0.137) were also affected by a large interaction effect of the Environment*WBV after 20 weeks of intervention.

**Table 3:** Inflammatory, bone turnover and metabolic biomarkers before and after the intervention (mean ± SD)

	Normoxia			Hypoxia			Normoxia + WBV			Hypoxia + WBV			PrePost*Env	PrePost*WBV	PrePost*Env*WBV
	Pre	Post	95%CI	Pre	Post	95%CI	Pre	Post	95% CI	Pre	Post	95%CI	F (effect size)	F (effect size)	F (effect size)
CRP (mg/L)	4.0 ± 2.0	4.1 ± 1.7	− 0.69–0.57	5.4 ± 0.4	3.6 ± 0.7**	1.38–2.27	3.2 ± 0.9	3.0 ± 1.2	− 0.29–0.63	3.0 ± 0.7	2.6 ± 0.9*	− 0.54–1.31	**12.267 (0.222)**^**++**^	**4.137 (0.088)**^**+**^	**7.789 (0.153)**^**++**^
IL-8 (pg/ml)	2.0 ± 1.0	1.9 ± 0.9	− 0.20–0.41	1.6 ± 0.5	1.5 ± 0.3	− 0.16–0.47	1.5 ± 0.4	1.7 ± 0.5	− 0.45–0.15	2.0 ± 0.3	1.8 ± 0.9	− 0.67–1.18	1.409 (0.032)	0.156 (0.004)	0.848 (0.019)
IL-10 (pg/ml)	1.5 ± 0.5	1.6 ± 0.5	− 0.39–0.29	1.6 ± 0.2	1.8 ± 0.4	− 0.24–0.13	1.6 ± 0.4	1.5 ± 0.4	− 0.11–0.33	1.5 ± 0.3	2.2 ± 0.2**	− 0.87–0.01	**6.796 (0.136)**^**+**^	2.378 (0.052)	**6.822 (0.137)**^**+**^
PINP (ng/ml)	75.9 ± 21.6	74.0 ± 18.5	− 8.12–12.05	77.6 ± 12.9	94.0 ± 10.6*	− 24.70–− 8.15	84.0 ± 17.4	101.1 ± 20.2**	− 31.69–− 2.51	73.2 ± 9.7	95.6 ± 17.3**	− 36.53–− 8.39	**4.071 (0.086)**^**+**^	**4.548 (0.096)**^**+**^	1.225 (0.037)
b-CTX (pg/ml)	115.0 ± 24.7	106.9 ± 27.5	− 5.9–22.3	134.7 ± 27.6	125.4 ± 27.2	− 12.22–30.82	130.4 ± 21.1	121.1 ± 25.5	− 4.30–42.9	113.8 ± 25.2	103.2 ± 23.1	− 8.3–29.6	0.158 (0.004)	0.431 (0.010)	0.266 (0.006)
Glucose (mg/dL)	101.2 ± 11.5	100.4 ± 10.2	− 2.12–3.77	88.5 ± 10.9	84.6 ± 9.8	− 4.11–11.92	96.5 ± 31.8	91.9 ± 18.11	− 6.85–16.09	97.5 ± 12.5	98.0 ± 13.1	− 2.86–2.01	0.073 (0.002)	0.005 (0.001)	1.242 (0.028)
Triglycerides (mg/dL)	76.7 ± 35.8	88.0 ± 46.01	− 29.61–7.02	73.9 ± 30.0	87.9 ± 54.3	− 41.35–13.35	75.2 ± 31.0	81.5 ± 29.5	− 19.40–6.79	95.8 ± 59	79.5 ± 28.6	− 13.47–46.04	0.991 (0.023)	3.118 (0.068)	1.604 (0.036)
CHO (mg/dL)	179.4 ± 23.0	189.6 ± 39.7	− 32.73–12.26	171.6 ± 35.3	176.1 ± 36.6	− 30.48–21.48	187.5 ± 23.2	183.8 ± 24.23	− 5.82–13.21	205.7 ± 34.2	218.0 ± 43.3	− 47.84–23.26	0.219 (0.005)	0.079 (0.002)	0.985 (0.022)

Values that reach statistical significance are in bold

*Env* environment factor; *WBV* whole-body vibration factor; *CRP* C-reactive protein; *IL-8* interleukin 8; *IL-10* interleukin 10; *PINP* N-terminal propeptide of type I procollagen; *b-CTX* C-terminal telopeptide of collagen; CHO: total cholesterol

*Within-group significant differences (pre–post). *p ≤ 0.05; **p ≤ 0.01

^+^Between-group significant differences. ^+^*p* ≤ 0.05; ^++^*p* ≤ 0.01

The values obtained in the functional fitness tests are shown in Table 4. There were significant increases in the chair stand and the front plank values after the intervention with WBV, both in normoxia and hypoxia. An interaction effect of WBV factor was observed in the values of chair stand (*F* = 4.122; *p* = 0.046; ES: 0.089) and front plank (*F* = 9.895; *p* = 0.003; ES: 0.187). The environment factor did not have any significant effect on the variables referring to functional fitness.

**Table 4:** Functional fitness tests before and after the intervention (mean ± SD)

	Normoxia			Hypoxia			Normoxia + WBV			Hypoxia + WBV			PrePost*Env	PrePost*WBV	PrePost*Env*WBV
	Pre	Post	95%CI	Pre	Post	95%CI	Pre	Post	95%CI	Pre	Post	95%CI	F (effect size)	F (effect size)	F (effect size)
Chair stand (rep)	12.4 ± 2.4	13.2 ± 2.4	− 2.41–0.88	16.7 ± 3.1	16.5 ± 3.7	− 3.28–3.68	15.4 ± 2.4	16.9 ± 2.7*	− 2.29–− 0.47	13.1 ± 1.0	16.1 ± 2.1*	− 4.85–− 1.15	0.112 (0.003)	**4.122 (0.089)**^**+**^	1.759 (0.039)
Arm curl (rep)	15.1 ± 4.0	15.7 ± 4.8	− 2.32–1.02	18.3 ± 4.2	19.4 ± 4.9	− 3.29–1.09	16.1 ± 3.3	17.5 ± 3.9	− 2.29–− 0.47	15.5 ± 3.4	17.5 ± 3.2	− 5.72–− 0.27	0.033 (0.001)	1.285 (0.029)	0.066 (0.002)
6MWT (m)	545.0 ± 66.3	536.0 ± 57.2	− 0.64–49.50	548.0 ± 32.9	551.0 ± 28.9	− 64.53–3.93	548.0 ± 52.4	543.0 ± 48.7	− 14.29–25.13	523.0 ± 55.3	540.0 ± 36.6	− 53.52–19.67	3.254 (0.069)	0.883 (0.020)	0.364 (0.008)
8 ft. up and go (s)	5.9 ± 1.0	5.7 ± 0.9	− 0.05–0.49	6.0 ± 0.3	6.2 ± 0.4	− 0.33–0.11	6.1 ± 0.9	6.3 ± 0.8	− 0.61–0.14	6.1 ± 0.5	6.0 ± 0.7	− 0.29–0.51	0.092 (0.003)	0.336 (0.008)	3.759 (0.077)
Sit and reach (cm)	− 1.59 ± 8.2	2.24 ± 14.6	− 8.86–1.21	− 4.0 ± 14.5	− 1.5 ± 15.4	− 8.99–3.99	− 1.4 ± 10.7	0.2 ± 11.9	− 5.09–2.63	2.4 ± 3.5	6.4 ± 5.4	− 11.39–3.39	0.075 (0.002)	0.043 (0.001)	0.603 (0.014)
Hand grip (kg)	27.5 ± 9.3	27.4 ± 9.6	− 1.43–1.57	27.0 ± 6.2	27.9 ± 6.6	− 2.26–1.82	33.2 ± 13.5	32.0 ± 12.08	− 0.20–2.50	26.8 ± 9.8	27.9 ± 8.2	− 2.95–0.78	2.452 (0.054)	0.017 (0.001)	1.452 (0.033)
Front plank (s)	45.9 ± 20.6	48.2 ± 21.2	− 11.93–7.44	40.0 ± 7.4	40.2 ± 8.5	− 0.07–8.07	53.5 ± 12.9	70.0 ± 22.7**	− 27.14–− 5.92	46.8 ± 12.1	65.5 ± 8.0**	− 28.41–− 2.04	0.001 (0.001)	**9.895 (0.187)**^**++**^	0.160 (0.004)

Values that reach statistical significance are in bold

*Env* environment factor; *WBV* whole-body vibration factor; *6MWT* 6-min walking test

*Within-group significant differences (pre–post). *p ≤ 0.05; **p ≤ 0.01

^+^Between-group significant differences. ^+^*p* ≤ 0.05; ^++^*p* ≤ 0.01

## Discussion

The initial hypothesis of the study was partially fulfilled since a 20-week combined training of IH and WBV for older adults led to an improvement of the inflammatory status (CRP and IL-10), as well as an increase of muscle mass, BMC, and PINP (bone formation biomarker). There was also an increase of physical performance in both the chair stand and front plank tests, although these improvements seem to be due to WBV training without observing an added effect of IH. Moreover, IH and WBV did not cause any significant change in fat mass or metabolic parameters (glucose, triglycerides, and CHO).

Aging has been associated with a state of chronic low-grade inflammation in which the older adults have high circulating levels of CRP and pro-inflammatory cytokines [34]. Against this, a recent systematic review has concluded that WBV could be useful in the management of inflammatory conditions, both in healthy populations and in individuals with chronic diseases [35]. An 8-week WBV training program on a vibration platform (4 mm and 20–35 Hz; 2 sessions/week) led to a significant decrease in CRP and an increase in IL-10 mRNA content [13]. Similarly, exposure to moderate levels of IH was effective in lowering circulating CRP levels [18] and increase levels of the anti-inflammatory cytokine IL-10 [36]. These previous results are consistent with those obtained in the present study, in which IH had a beneficial effect on the inflammatory status of the participants, producing a positive interaction effect when combined with WBV. The combined effect of IH and WBV could modulate the immune response, which seems to be mediated by a downregulation of the toll-like receptors (TLR2 and TLR4) [13]. This modulation would exert an anti-inflammatory and tissue-protective effects, suppressing pro-inflammatory mediators such as TNF-α and IL-4 [37], and increasing the IL-10 production by B cells [36].

Regarding the bone health, it is observed that both environment factor and WBV factor had a positive effect on PINP levels, which is a biomarker of bone formation. A 24-week (3 sessions/week) intervention consisting of 45-min sessions of passive normobaric IH exposures also showed a significant increase of PINP in older people [18]. IH exposure could activate different genes involved in bone remodeling, as vascular endothelial growth factor (VEGF), erythropoietin (EPO) ,and osteoprotegerin [38, 39], modulating the bone turnover biomarkers. Likewise, regarding WBV, a previous study also obtained improvements in bone turnover biomarkers after an 8-week intervention of low-frequency (12 Hz) and low-magnitude WBV (0.3 g) [40]. In contrast, a recent systematic review with meta-analysis has concluded that WBV had no significant effect on either bone formation biomarkers or bone resorption biomarkers [41]. This lack of consistency of results after a WBV intervention could be explained by the wide variety of protocols used, since the time course, dose, and frequency of the vibration could be determining factors to elicit significant changes in bone turnover biomarkers. On the other hand, WBV training alone did not seem to be enough stimulus to modify BMC. In this sense, the cumulative dose of WBV has been positively related with improved bone mass [42], having been proposed a minimum number of 108 training sessions to obtain significant differences at the BMD level in older people [12]. In our study, the number of sessions was only 60 sessions, and it is also necessary to highlight that the intake of calcium and vitamin D (boosters of bone formation) of our participants was below RDI. However, a significant increase in BMC was observed in HWBV. The addition of the hypoxic stimulus to the vibratory stress could stimulate bone growth, as it has been stated in a previous study by our research group [28]. The increased production of nitric oxide levels caused by the hypoxic environment would reduce bone resorption by inhibiting osteoclast formation and resorptive function of mature osteoclasts [43].

The different interventions did not cause changes in the variables of weight, BMI, fat mass or muscle mass, except for HWBV in which a significant increase in muscle mass was observed. It is worth to note that changes in body composition depend on various factors, such as the type of intervention, the nutritional and caloric intake, or the individual adaptive response [44, 45]. Given that the participants were not forced to have a specific diet throughout the intervention, the lack of significant changes in body composition parameters could be influenced by this issue. Regardless, previous systematic reviews have concluded that WBV training alone may not be a sufficient stimulus to increase lean mass in postmenopausal women and older adults [46, 47]. Indeed, in our research, it was necessary to add the synergistic effect of IH to WBV to achieve significant changes in muscle mass values. Hypoxic conditioning has been proposed as a therapeutic modality to mitigate the sarcopenia and to improve muscle hypertrophy and muscle function [19, 48]. A moderate hypoxic environment (10–15% FiO_2_) may promote muscle differentiation and hypertrophy by increasing the expression of proteins associated with muscle cell differentiation (myogenin and the expression of mTOR) [49]. However, the only previously published study on combined WBV and IH training concluded that a 36-session intervention (2 sessions/week) caused no change in leg lean mass of older adults. [29]. These different results could be explained by the duration of the intervention (60 vs 36 training sessions), as well as by the number of WBV exercises proposed (4 vs 1) and the cumulative dose of WBV and IH that were significantly greater.

WBV training, both under hypoxic and normoxic conditions, had a significant positive effect on the physical performance obtained in the chair stand and front plank tests. Previous studies have stated that WBV training improved significantly the physical fitness of the older people (including balance, strength, agility, walking speed and endurance) [50], especially in older people with a low level of muscle strength [51]. The vibration leads to alterations in the length of the muscle–tendon complex and muscle spindles that activates muscle contraction through reflex muscular activity [52]. Although there has been a general improvement in the functional fitness of the participants, we speculate that it has only reached statistical significance in those two tests because the four exercises performed during the intervention focused on the muscles that were later evaluated with the chair stand and front plank test. IH did not cause any synergistic effect added to the physical performance obtained with the WBV training. A recent systematic review has proposed a limited effect of passive/active IH on functional fitness of healthy older adults compared to normoxic intervention, indicating that only long-term interventions with a high hypoxic dose (severity and exposure time) could provide an added effect [53].

The study had several limitations. The experimental design did not have a sham WBV group since NOR and HYP groups were seated without receiving any vibration. The participants were only blinded to the hypoxic or normoxic condition. In addition, the diet and caloric intake were not monitored during the intervention. At the beginning of the intervention, participants were asked to continue with their usual diet and only a record of caloric intake was carried out. Finally, the use of imaging techniques (magnetic resonance and computed tomography) to evaluate the muscle cross-sectional area, regional skeletal muscle and bone geometric properties would have provided more detailed information on changes in body composition and muscle quality (strength per unit tissue).

## Conclusion

In conclusion, the results showed that combined WBV and IH training in older adults had beneficial effects on inflammatory status, bone health, and body composition. This combined training could be used as a therapy to prevent the impairment of health-related outcomes associated with aging. However, more research on WBV and IH is needed to confirm the beneficial effects observed in this study, as well as the mechanisms responsible for the adaptations.

## Data Availability

Data available on request from the authors.
